# Supplementary material for: Bioaccumulation of Blood Long-Chain Fatty Acids during Hemodialysis
Source: Metabolites. 2022 Mar 21;12(3):269. doi: 10.3390/metabo12030269 (PMC8949028; doi:10.3390/metabo12030269)
Supplement: Supplementary file 1 [file metabolites-12-00269-s001.zip › metabolites-1626258-SI.pdf]

Table S1 Clinical parameters of patients (n=12).

Parameters	Patients
Glucose (60-110 mg/dL)	115.8 ± 36.7
Total cholesterol (< 200 mg/dL)	221.8 ± 181.2
LDL-cholesterol (< 130 mg/dL)	101.2 ± 30.7
HDL- cholesterol (>35 mg/dL)	42.4 ± 9.9
Triglycerides (<200 mg/dL)	151.0 ± 77.4

Notes: Data are presented as mean ± SD.

Table S2. Effect of hemodialysis on polyunsaturated fatty acid ratios in venous plasma and RBC in the CKD patients before (Pre-HD) and at cessation (Post-HD) of hemodialysis (n=12 each).

Ratio	pre-HD Venous	post-HD Venous	p value, t test (# paired Wilcoxon test)
**Total-Plasma**			
DHA+EPA/AA	0.7514 (0.6267 –0.8752)	0.6516 (0.5946 –0.8166)	0.127
EPA/AA	0.0983 (0.0700 –0.1537)	0.0806 (0.0715 –0.1303)	0.286#
DHA/AA	0.6359 (0.5586 –0.6793)	0.5780 (0.5115 –0.6609)	0.182#
DHA/EPA	6.5543 (5.1800 –7.4374)	6.5892 (5.8563 –7.3498)	0.793
n-3/n-6	0.1834 (0.1511 –0.2118)	0.1651 (0.1541 –0.2176)	0.81
**Total-RBC**			
Omega-3 quotient	11.3752 (7.7099 –12.1713)	11.3738 (8.3040 –12.5440)	0.676
DHA+EPA/AA	0.6547 (0.5208 –0.9044)	0.7027 (0.6151 –0.8107)	0.826
EPA/AA	0.0341 (0.0295 –0.0575)	0.0421 (0.0335 –0.0512)	0.404
DHA/AA	0.6202 (0.5050 –0.8469)	0.6605 (0.5833 –0.7487)	0.89
DHA/EPA	15.2611 (14.5957 – 21.7166)	17.3803 (13.4656 – 20.3596)	0.433#
n-3/n-6	0.4098 (0.3518 –0.5048)	0.4095 (0.3878 –0.4616)	0.701

Notes: Median (IQR)

Table S3. Effect of hemodialysis on the ratio of desaturase and peroxisome function in venous plasma and RBC in the CKD patients before (Pre-HD) and at cessation (Post-HD) of hemodialysis (n=12 each).

Ratio	pre-HD Venous	post-HD Venous	p value, t test (# paired Wilcoxon test)
**Total-Plasma**			
DHA/DPA	8.9873 (7.0171 –10.7715)	9.0468 (6.5445 –13.8399)	0.468
C20:4 n-6/C20:3 n-6 (Δ5 SCD)	3.5725 (3.2953 –4.0303)	3.9063 (3.3146 –4.7591)	0.088
C18:3 n-6/C18:2 n-6 (Δ6 SCD)	0.0149 (0.0102 –0.0185)	0.0167 (0.0112 –0.0204)	0.556#
C16:1 n-7/C16:0 (Δ9 SCD)	0.0880 (0.0808 –0.1015)	0.0859 (0.0763 –0.0921)	0.053
C18:1 n-9/C18:0 (Δ9 SCD)	5.7640 (4.6422 –7.0182)	6.0049 (4.9686 –6.2736)	0.599
**Total-RBC**			
DHA/DPA	5.0147 (3.5409 –5.9286)	4.9854 (4.3682 –5.8391)	0.877
C20:4 n-6/C20:3 n-6 (Δ5 SCD)	8.0572 (6.6672 –10.3164)	8.1449 (7.5088 –10.8905)	0.814#
C18:3 n-6/C18:2 n-6 (Δ6 SCD)	0.0108 (0.0067 –0.0131)	0.0110 (0.0068 –0.0136)	0.429
C16:1 n-7/C16:0 (Δ9 SCD)	0.0437 (0.0328 –0.0569)	0.0479 (0.0380 –0.0574)	0.371
C18:1 n-9/C18:0 (Δ9 SCD)	1.2582 (0.9138 –1.5227)	1.3379 (1.1765 –1.4844)	0.275

Notes: Median (IQR)
